# A simple behavioral evaluation test of human face identity recognition with natural images validated with the case of prosopagnosia PS

**DOI:** 10.1038/s41598-025-27165-9

**Published:** 2025-12-04

**Authors:** Angélique Volfart, Caroline Michel, Bruno Rossion

**Affiliations:** 1 Department of Behavioural and Cognitive Sciences, Faculty of Humanities, Social and Educational Sciences, Institute of Cognitive Science and Assessment, University of Luxembourg, Esch-sur-Alzette, Luxembourg; 2 Psychological Sciences Research Institute (IPSY), Université de Louvain, Louvain-la-Neuve, Belgium; 3 Université de Lorraine, CNRS, IMoPA UMR 7365, Pavillon Krug (1er étage-entrée CC-1), Hopital Central, Nancy, 54000 France; 4 Service de Neurologie, CHRU Nancy-University Hospital of Nancy, 29 Av. du maréchal de Lattres de Tassigny, Nancy Cedex, 54035 France

**Keywords:** Face identity recognition, Natural images, Prosopagnosia, Single case, PS, Human behaviour, Neurological disorders

## Abstract

Behavioral evaluation of face identity recognition (FIR) ability has been performed with images for decades but remains subject to challenges regarding the stimuli and tasks used as well as the measured variables. Here we validate two simple nonverbal tests of FIR with ambient natural face images in neurotypical individuals and the well-documented brain-damaged case of prosopagnosia PS. In the first task, participants must select a famous identity among 3 simultaneously presented pictures. While neurotypical individuals scored almost at ceiling, PS was at chance level. In contrast, she was flawless at the same task with written names. The second task requires matching different views of familiar or unfamiliar faces for their identity against one distractor face, at upright or inverted orientation. While performing well above chance level, PS was significantly impaired and slowed down compared to neurotypical controls. Moreover, unlike controls, she showed no advantage for familiar or upright faces. Altogether, these simple behavioral FIR tests present with numerous advantages in terms of validity (i.e., natural images, lack of response biases, familiarity and inversion effects) and sensitivity (i.e., high contrast between patients and controls), making them particularly advantageous to rapidly evaluate the integrity of this function in normal and pathological populations.

**Supplementary Information:**

The online version contains supplementary material available at 10.1038/s41598-025-27165-9.

## Introduction

The ability to identify individual people from their face, i.e., face identity recognition (FIR), is a key brain function in humans, arguably the ultimate recognition function in our species^1^. FIR has been studied experimentally with face pictures since the 1960s, originally with neuropsychological tests developed to assess this function in brain-damaged patients^2–6^. Over the past three decades, the number of behavioral tests developed to assess FIR has grown significantly (^7^ for review). These tests, such as the widely used Cambridge Face Memory Test (CFMT^8^, aim at maximizing inter-individual variability and test-retest reliability in the normal population, and have generally been successful at reaching these objectives. To achieve this, however, the tests are intentionally challenging. This difficulty often stems from the involvement of general cognitive processes and/or response uncertainty, and the use of stimuli that are artificially hard to discriminate based on their identity. For example, the CFMT requires participants to explicitly learn and retain pictures of novel face identities, before having to select these learned faces from new distractors presented side-by-side under various formats. This makes this test relatively complex in terms of instructions in addition to involving short- and long-term visual memory, selective and spatial attention, decision making, etc. Moreover, FIR tests usually labelled as ‘perceptual’ (as opposed to so-called ‘memory’ tests), which involve matching or discriminating simultaneously presented face pictures based on their identity, are made difficult by typically using stimuli with masked or cropped external features (e.g., removing hair and ears), or highly similar distractors selected or generated artificially (e.g., selection of the distractor faces based on the worst performing trials, or morphs between target and non-target faces) (e.g., CFPT^9^, GFMT^10^, USCFPT^11^, CFT^12^. Some tests also introduce biases in response selection (e.g., same/different judgments: GFMT^10^, EFCT^13^, KFMT^14^. While these measures successfully produce high inter-individual variability and reliability scores, they arguably lack simplicity, sensitivity and validity for daily use in clinical neuropsychology^7^.

In the same vein, while several tests have been developed to assess famous face recognition, most of them also rely on edited or cropped images that remove external facial features such as hair and ears (e.g.^15–18^), and are typically presented in greyscale or black-and-white formats (e.g.^19–23^). These design choices, while aiming to control for low-level visual cues, reduce ecological validity and may increase task difficulty in ways that are not representative of real-world face identity recognition. Additionally, most of the above-mentioned tasks require verbal naming or explicit retrieval of person-related semantic information to some extent, which can be problematic in clinical populations with language impairments and also involve laborious scoring procedures.

In two recent studies performed in the context of transient direct electrical stimulation (DES) to face-selective brain regions that are key for FIR, we introduced simple tasks in which the stimulated patients had to choose a natural ambient face image of a celebrity among 3 options (Task 1) and match either familiar or unfamiliar faces for their identity against one distractor face (Task 2)^24,25^. In both studies, we found the tasks to be ideal in a highly-constrained clinical context for their sensitivity (i.e., maximizing contrast between the patient’s performance with and without stimulation), simplicity, and rapidity of assessment. However, due to specific testing constraints, these tasks were only composed of a few items, administered manually (see e.g., videos in^24^).

Here we provide validation of an unpublished full electronic version of the two tasks, with a relatively large (fixed) number of items. It is important to note that the present study is not intended to provide a widely usable test without adaptations, or normative data or diagnostic cutoffs, but rather to demonstrate the diagnostic value of these tasks in distinguishing between typical and impaired FIR performance. In the present study, we compare performance of neurotypical individuals at these FIR tasks to a well-known case of prosopagnosia (PS) following brain damage, arguably the most-documented case in the scientific literature (first report^26^, recent reviews in^27,28^). In both tasks, we maximize ecological validity by using natural images that vary in unsystematic ways (ambient faces, e.g.,^29–31^). Yet, even for unfamiliar faces, the type of task (forced-choice) and stimulus selection is well-balanced to make it relatively easy for neurotypical individuals and at the same time highly challenging for the case of prosopagnosia. Using both famous and unfamiliar faces also allows to tap into distinct but complementary components of FIR: while the face matching task mainly relies on the ability to discriminate faces based on their visual features for unfamiliar faces, familiar faces may additionally involve linking the perceived facial identity to semantic memory. In the familiarity selection task, this ability to link a face to its associated (semantic) information is not optional but essential, and the implementation of a name counterpart condition provides important information about the specificity of the deficit. Besides manipulating familiarity (i.e., famousness of faces here), we also compare performance at matching faces presented at upright and inverted orientations, a manipulation that was not performed in the DES studies referred to above. This simple stimulus manipulation, which preserves the physical difference between the faces presented at upright or inverted orientation, has long been known for leading to large and category-specific decreases of FIR performance in neurotypical observers^32^, in particular during face identity matching tasks (^33^, for review^34^. In contrast, brain-damaged patients with FIR impairments show less or no effect of inversion^35,36^, providing an additional useful marker of FIR ability. Finally, the tasks presented here involve simple forced-choice paradigms that are rapid to administer and score, and do not require any explicit verbal output. These features make the present FIR tasks particularly suitable for clinical neuropsychological assessment.

## Test 1: forced-choice familiarity selection

### Objectives

To develop a task targeting famous people recognition ability without verbal output and contrasting face and name recognition performance of the same identities to assess potential face-name dissociations.

### Materials and methods

#### Participants

*Younger participants.* Seventeen undergraduate participants (11 females; mean age 23.76 ± 3.07) were recruited. They were either French or Belgian, but were all tested at the University of Louvain in Belgium.

*PS.* Subject PS is a right-handed Swiss woman (born in 1950) who sustained head injury in 1992. This resulted in bilateral but asymmetrical lesions, primarily affecting the right inferior occipital gyrus and the left mid-fusiform gyrus (see^37^ for detailed analysis). Despite initial cognitive deficits, PS recovered well following neuropsychological rehabilitation. Her only persistent issue is a severe impairment at recognizing individuals by their face. Since the first report^26^, her case of prosopagnosia has been extensively studied and described over the last twenty years (about 40 published papers; see the extensive reviews in^27,28^, most recent papers^38,39^. PS never complained nor presented any difficulties at recognizing non-face objects^40^. Investigation of her visual abilities showed lower-range normal color vision, good visual acuity, and full visual field except for a small paracentral scotoma in the upper left quadrant^37^.

Patient PS was tested only once on each of the tasks. She completed the face familiarity task in June 2016 at age 65, and the name familiarity task in May 2017 at age 66.

*Age-matched controls.* Eight older controls (4 females; mean age 66.62 ± 3.07) were recruited.

All methods were carried out in accordance with relevant guidelines and regulations. All participants provided their written informed consent to participate in the experimental protocols as approved by the ethical committee of the Psychological institute of the University of Louvain.

#### Procedure

Experimental tasks were created and tested using E-Prime 2.0 (Psychology Software Tools, Pittsburgh, USA). Participants were seated in front of a computer in a quiet room, at about 50 cm from the screen. They were asked to respond to each task by pressing pre-determined keys on the keyboard.

*Face familiarity.* This task was created with color natural photographs of 150 face identities (27 females) collected from the internet. The image selection followed several guidelines to ensure consistency and minimize confounds. Accessories that could obscure facial features (e.g., sunglasses, hats) were excluded, although vision glasses were allowed when they did not interfere with the task. All images were cropped to include the full face, leaving part of the neck/beginning of the shoulders, and the majority of the hair visible. Natural backgrounds were retained, and effort was made to ensure that faces occupied a similar proportion of space across triplets and trials. Fifty images depicted famous identities (mostly French and international Caucasian celebrities: actors, singers, politicians, etc.) selected to match the cultural context and age range of subject PS and to ensure familiarity within French-speaking populations. The remaining 100 images were photographs of non-famous identities (celebrities unknown to the French-speaking community). Each famous identity was paired with two non-famous identities according to gender and general physical appearance (age, hair color, hairstyle). Face photographs were mostly full-front views, but with slight variations of head position and natural changes of facial expression and lighting conditions. Face images subtended about 8.47° (width) x 11.35° (height) of visual angle.

**Fig. 1:**
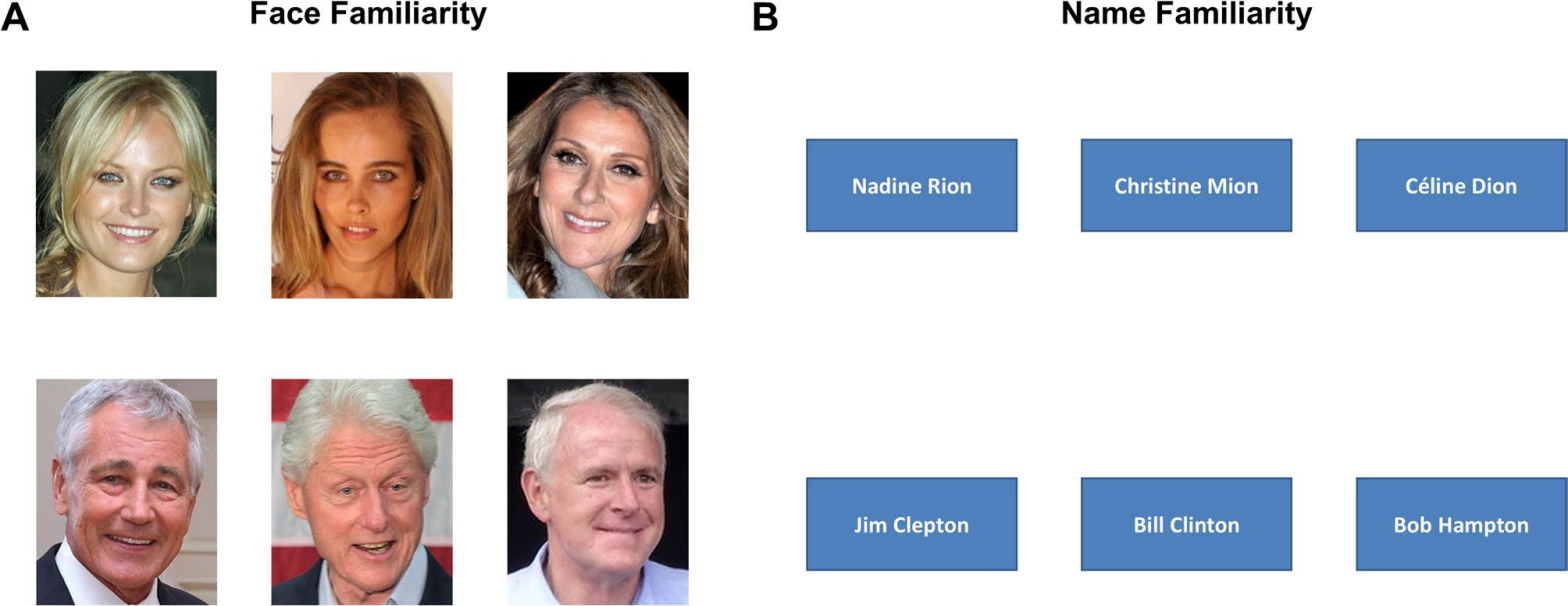
Illustration of trials presented during the face (**A**) and name (**B**) familiarity selection tasks. Note that face images displayed here are not the exact same images as presented in the original tasks due to copyrights. Equivalent images with copyright agreements (CC-BY) are represented here. For license information, Malin Akerman: https://flic.kr/p/5koy3m (CC BY 2.0), David Shankbone; Isabel Lucas: https://flic.kr/p/bHbQ4a (CC BY-SA 2.0), Eva Rinaldi; Céline Dion: https://commons.wikimedia.org/w/index.php?curid=22910240 (CC BY-SA 3.0), Hubert Burda Media; Chuck Hagel: https://flic.kr/p/pC2677 (CC BY 2.0), Gobierno de Chile; Bill Clinton: https://flic.kr/p/EBUeYB (CC BY-SA 2.0), Gage Skidmore; Tom Barrett: https://flic.kr/p/vhmKXg (CC BY-SA 2.0), jchapwiesky.

Each trial consisted of three face photographs simultaneously presented on the screen, side-by-side: one famous identity and its two non-famous distractor identities (Fig. 1A). Position of the famous identity was counterbalanced across trials. Participants had to find which face identity was famous by pressing “1”, “2”, or “3” on the keyboard according to the location of the face on the screen (either left, middle or right). Triplets of faces remained on the screen until the participant’s response. A blank screen was presented for 1000ms between each trial. The experiment consisted of one block of 50 trials and the order of trials was randomized for each participant.

*Name familiarity.* This task was similar to the face familiarity task, except that each famous face was replaced by its corresponding written name and non-famous distractors were replaced by fictional phonologically-similar names (Fig. 1B). Famous identities were the same in the face and name familiarity tasks. Names had a size in visual angle comprised between 2.36° and 8.24° (width) and between 0.59° and 1.90° (height).

#### Statistical analyses

Each E-Prime datafile was analysed individually to calculate accuracy scores and correct RT for each trial type. For each participant, correct RT outliers (> 3 SD) were removed from the calculation of the mean correct RT.

Differences in performance according to trial types in undergraduate and age-matched controls were separately analysed with repeated-measures ANOVAs. These analyses were performed with the SPSS software (IBM SPSS Statistics, Version 20.0; Armonk, NY: IBM Corp.).

Subject PS’s performance was compared with her age-matched controls using Crawford & Howell two-tailed t-tests for single-case comparisons (Singlims software^41^). A False Discovery Rate (FDR) correction was applied on p-values to control for multiple comparisons when comparing PS and her controls. We tested for dissociations in PS compared to her controls with Crawford & Howell revised standardized difference tests (RSDT; Dissocs software^42^).

### Results

We first report the data obtained in younger participants, and then on subject PS vs. her age-matched controls. The full distribution of data in controls is presented in Supplementary Figure S1. All data is available in Supplementary Tables 1 and 2.

#### Younger participants

Participants performed at ceiling on both face and name familiarity tasks (Table 1), indicating that all famous people represented in these tasks were well-known to this population. A paired t-test showed a trend for higher accuracy scores with names than faces (t(16) = 1.986, *p* = 0.064), and significantly faster RTs for names than faces (t(16) = 3.179, *p* = 0.006).

**Table 1:** Performance on the face and name familiarity tasks in each category of participants.

Test measure	Trial type	Young controls (*n* = 17)	Age-matched controls (*n* = 8)	PS
Accuracy (%)	Face	94.47 (5.08)	97.5 (3.16)	36
	Name	97.06 (4.13)	98.5 (3.51)	100
Correct RT (ms)	Face	1533 (354)	1930 (555)	13,566
	Name	1324 (293)	1646 (332)	4381

Results are displayed as mean (standard deviation).

#### PS and age-matched controls

**Fig. 2:**
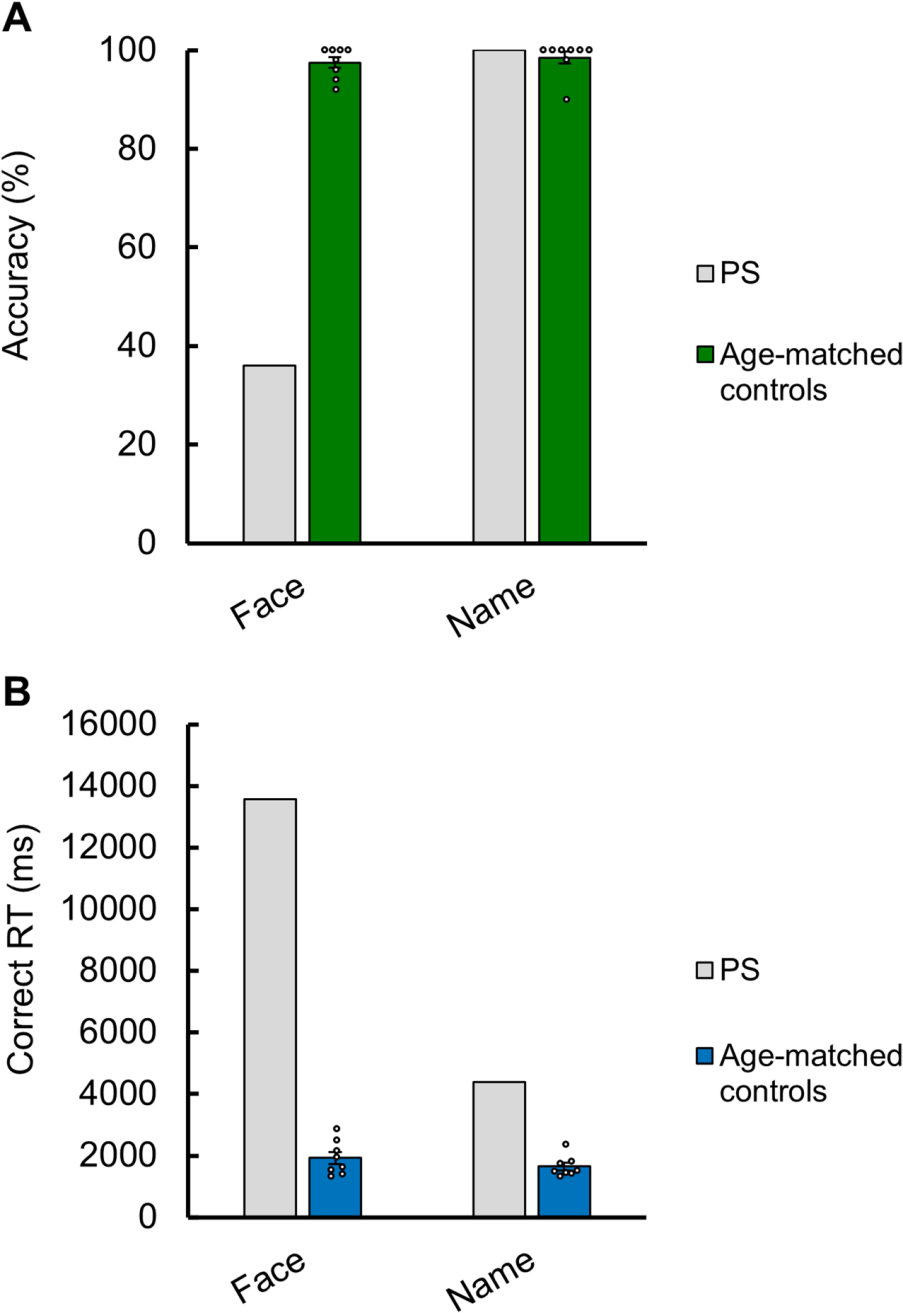
Mean accuracy (**A**) and correct response times (**B**) of PS and 8 control participants on the face and name familiarity tasks. Black and white dots represent individual scores. Error bars represent standard errors.

Results of PS and age-matched controls on the face and name familiarity tasks are displayed in Fig. 2 and reported in Table 1.

Similarly to younger participants, neurotypical individuals matched in age with PS also performed the task at ceiling for both faces and names (Table 1), without any difference between the two tasks (t(7) = 0.642, *p* = 0.542) (Fig. 2A). While their mean correct RT were faster for names than faces, the difference was not significant (t(7) = 1.316, *p* = 0.230) (Fig. 2B).

In stark contrast with her age-matched controls, PS was at chance level for the face familiarity selection task (36%; not significantly different from chance level at 33.3%; χ^2^ = 0.178, *p* = 0.673), but scored perfectly for the same task on written names (100%). The difference between her face and name scores was highly significant (χ^2^ = 47.059, *p* < 0.001). When she responded correctly, she was also much slower for faces than names (t(19.315) = 6.758, *p* < 0.001, non-equal variance t-test).

PS was significantly slower than her controls both on faces (about 7 times slower) and names (about 2.7 times slower) (face: t = 19.767, FDR-corrected *p* < 0.001; name: t = 7.767, FDR-corrected *p* < 0.001). The RSDT test assessing evidence for face-name dissociation in PS as compared to her age-matched controls highlighted a significant difference on both accuracy scores and correct RTs (accuracy: t = 10.342, FDR-corrected *p* < 0.001; RT: t = 7.312, FDR-corrected *p* < 0.001) but fulfilled the criteria for a classical face-name dissociation on accuracy only.

### Discussion

While neurotypical individuals, younger or older, performed the face familiarity selection task at ceiling, PS performed at chance level in that task. That is, the contrast between the patient and the normal controls is maximal. While this is expected given PS’s prosopagnosia, her chance level performance is still noticeable because she can sometimes recognize a famous or personally familiar face identity presented as a single picture, especially with iconic images or highly familiar faces shown in typical views^26,43^ and /or when she is aware of the pool of familiar faces presented (e.g., children of the kindergarten where she used to work^36,43^. Here, due to careful selection of images and distractors, without any editing, PS is just unable to use any sort of cue from, e.g., facial expression or the background of the natural images, to guess the level of famousness of the face identities displayed, even when taking close to 15 s/triplet on average to perform the task. Importantly, this failure cannot be attributed to her lack of visual knowledge of these famous personalities, as she has always regularly watched national and international news on television, series, sport events, etc., as well as reading celebrity magazines. Moreover, and importantly she was flawless at performing the same selection task on written names. While she was slower at selecting the correct (famous) names compared to normal controls, she was more than three time faster for names as compared to faces, while age-matched controls did not show significant difference in terms of material. While PS is able to read, her relative slowing down for written names is expected for two reasons that are independent of people identity recognition. First, due to brain damage, she has a left paracentral scotoma^26,37^ typical of cases of prosopagnosia^44,45^ that prevents her from seeing part of the written word upon central fixation. Second, her brain damage in the left ventral occipito-temporal cortex is in the vicinity of the visual word form area (VWFA), which may slow down her reading ability^27^.

Overall, the simple famous face selection task with triplets allows to rapidly evaluate famous face and name knowledge, in a limited amount of time (< 5 min), without requiring a verbal output, i.e., naming, which is particularly challenging and can be affected in neurotypical observers and many clinical conditions even when the underlying semantic knowledge is preserved.

## Test 2: forced-choice face identity matching

### Objectives

To develop a face identity matching task with natural images, with both familiar and unfamiliar faces (to assess the face familiarity effect) and upright and inverted orientations (to assess the face inversion effect), two key constructs of human face identity recognition.

### Materials and methods

#### Participants

*Younger participants.* Eighteen undergraduate participants (13 females; mean age 23.17 ± 2.79) were recruited for the face identity matching task. They were either of French or Belgian nationality, and were all tested at the University of Louvain in Belgium.

*PS.* PS was 66 years old when she completed the face matching task in May 2017.

*Age-matched controls.* Seven age-matched controls (6 females; mean age 67.86 ± 3.62) were recruited for the face identity matching task.

All methods were carried out in accordance with relevant guidelines and regulations. All participants provided their written informed consent to participate in the experimental protocols as approved by the ethical committee of the Psychological institute of the University of Louvain.

#### Procedure

Experimental tasks were created and tested using E-Prime 2.0. Participants were seated in front of a computer in a quiet room, at about 50 cm from the screen. They were asked to respond to each task by pressing pre-determined keys on the keyboard.

The famous and non-famous face simultaneous matching task at upright and inverted orientations (face matching) was created with 132 color natural face photographs collected from the internet. Guidelines for image selection were the same as for the familiarity selection task. The image set included 44 photographs of famous (*n* = 22, 4 females) or non-famous (*n* = 22, 5 females) face targets, 44 different photographs of these same target identities, and 44 photographs of other famous (*n* = 22, 4 females) or non-famous (*n* = 22, 5 females) face identities (i.e., distractors). Famous identities were mostly French and international Caucasian celebrities (actors, singers, politicians, etc.), while non-famous identities were celebrities from other countries, unknown among the French-speaking community. Targets and their respective distractors were matched according to gender, familiarity (famous or not), and general physical appearance (age, hair color, hairstyle). Face photographs mostly represented full-front views, but with slight variations of head position, under natural changes of facial expression and lighting conditions. Face images subtended about 10.59° (width) x 13.00° (height) of visual angle.

**Fig. 3:**
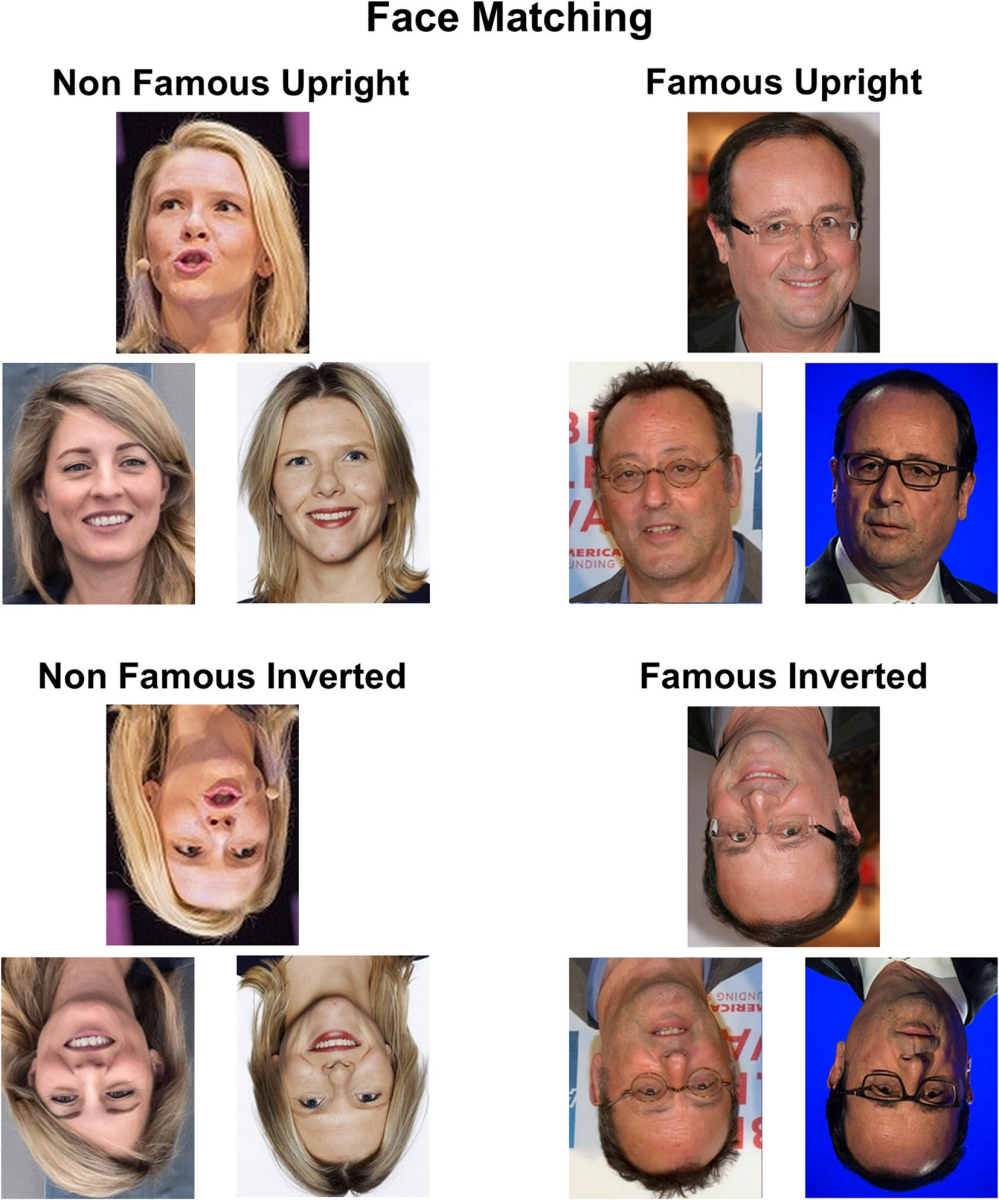
Illustration of trials presented during the face matching task. Note that face images displayed here are not the exact same images as presented in the original tasks because of copyrights. Equivalent images with copyright agreements (CC-BY) are represented here. For license information, Sylvi Listhaug: https://flic.kr/p/DJAdYE (CC BY 2.0), Kommunesektorens organisasjon; Wikimedia Commons (CC BY 3.0), FrPMedia; Mélanie Joly: https://flic.kr/p/2nN386y (CC BY-SA 2.0), Richard Koek; François Hollande: Wikimedia Commons (CC BY 2.0), A. Bouirabdane; Wikimedia Commons (CC BY-SA 3.0), C. Truong-Ngoc; Jean Reno: https://flic.kr/p/7XYrq2 (CC BY 2.0), David Shankbone.

Each trial consisted of three face photographs simultaneously presented on the screen, with the target face presented on top of two probes (Fig. 3). One probe was the other face photograph of the target identity, while the other probe was the face distractor. Participants had to find which of the probe corresponded to the same face identity as the target face. They were asked to answer by pressing the button “S” for “left” or “L” for “right” according to the location of the probe on the screen. Triplets of faces remained on the screen until the participant’s response and a blank screen was presented for 1000ms between each trial.

Each trial was presented at upright and upside-down (each image rotated at 180°) orientations, for a total of 44 famous trials and 44 non-famous trials. The experiment was divided into 4 blocks of 22 trials with a pause between each block. Each block included 11 famous and 11 non-famous trials, with half of the trials presented upright and the other half inverted. The same identity was never repeated in both orientations within the same block. Trials were presented in a randomized order within each block and blocks were always presented in the same order. Orientation was counterbalanced across identities: for example, of the 22 non-famous identities, 11 were first presented upright and 11 first presented inverted. The left/right position of the target face was also counterbalanced across trials within blocks.

#### Statistical analyses

Analyses were performed as in Test 1. Given that accuracy and RT data was not normally distributed, within-subject and within-item test-retest reliability was computed using Spearman’s rank-order correlations in JASP (version 0.95.1; https://jasp-stats.org/).

### Results

We first report the data obtained in the younger participants, then on PS vs. her age-matched controls. The full distribution of data in controls is presented in Supplementary Figure S2. All data is available in Supplementary Tables 3–5.

#### Younger participants

**Fig. 4:**
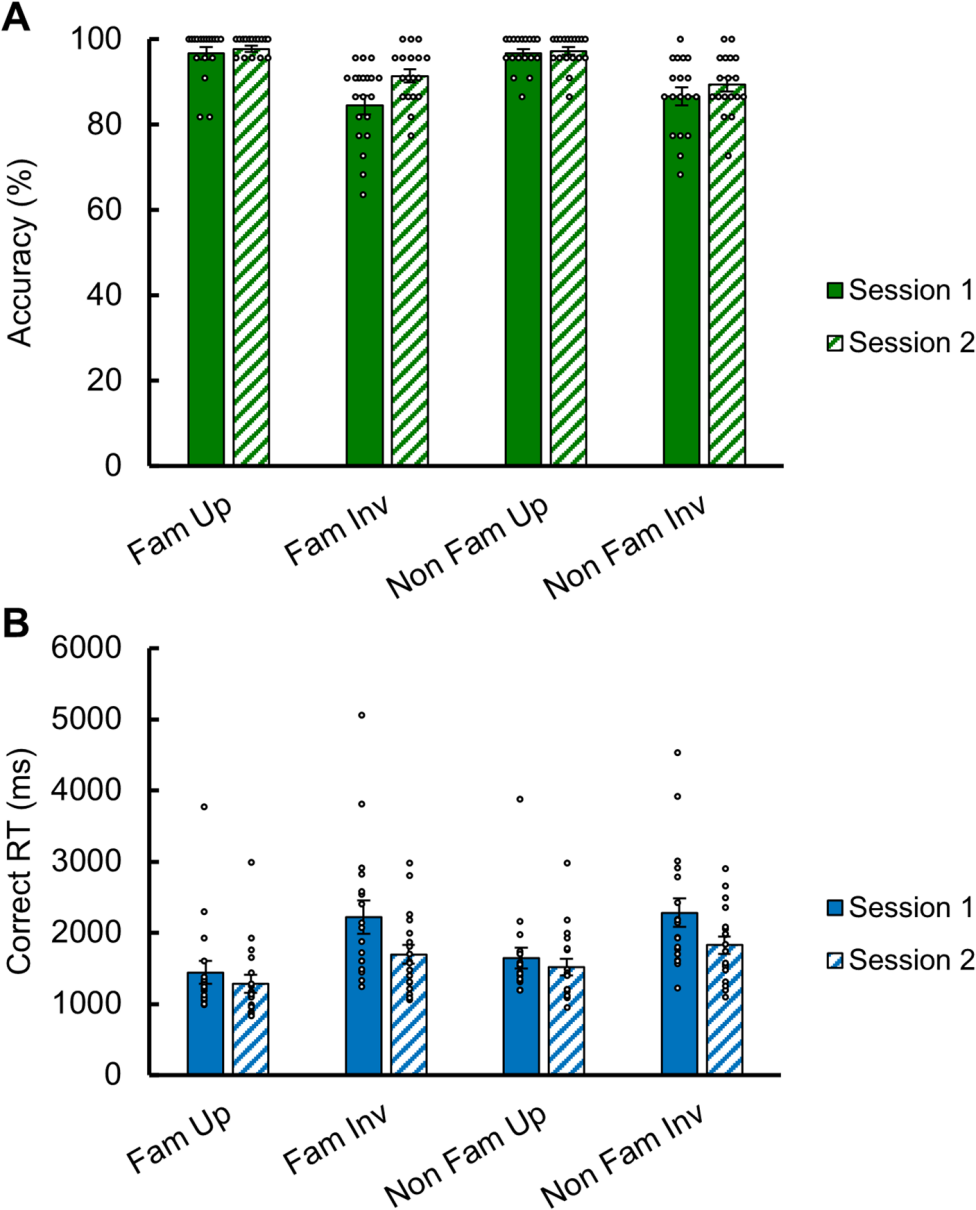
Mean accuracy (**A**) and correct response times (**B**) of 18 young participants at the face identity matching test across two testing sessions. Black and white dots represent individual scores. Error bars represent standard errors.

Undergraduate participants were tested twice consecutively with the face identity matching task to assess reliability. This task took about 5 to 10 min to be completed (including breaks). Results are presented in Table 2 and Fig. 4.

**Table 2:** Performance of undergraduate participants on the forced-choice face identity matching task.

Test measure	Testing session	Trial type	Performance (*n* = 18)
Accuracy (%)	1st	Fam Up	96.72 (6.00)
		Fam Inv	84.60 (9.51)
		Non Fam Up	96.73 (4.06)
		Non Fam Inv	86.64 (8.89)
	2nd	Fam Up	97.74 (3.21)
		Fam Inv	91.43 (6.41)
		Non Fam Up	97.24 (3.85)
		Non Fam Inv	89.41 (6.98)
Correct RT (ms)	1st	Fam Up	1447 (667)
		Fam Inv	2223 (979)
		Non Fam Up	1643 (615)
		Non Fam Inv	2285 (861)
	2nd	Fam Up	1285 (531)
		Fam Inv	1701 (560)
		Non Fam Up	1522 (504)
		Non Fam Inv	1831 (521)

Results are displayed as mean (standard deviation).

We first report the results of the first test administration (testing session 1). Overall, participants performed almost at ceiling, both for familiar and unfamiliar upright faces (Fig. 4A). They were on average 11.11% (± 7.52) more accurate for upright than inverted faces, irrespective of face familiarity. A two-way repeated measures ANOVA performed on accuracy with *Familiarity* (famous, non-famous), and *Orientation* (upright, inverted) as main factors showed only a significant effect of *Orientation* (F(1,17) = 39.210, *p* < 0.001; upright > inverted faces). There was no effect of *Familiarity* on accuracy scores (F(1,17) = 0.367, *p* = 0.553), nor any interaction effect (F(1,17) = 0.488, *p* = 0.494).

On correct RTs (Fig. 4B), participants were on average 709ms (± 500) faster for upright than inverted faces, irrespective of face familiarity. A two-way repeated measures ANOVA performed on correct RTs showed both a significant effect of *Familiarity* (F(1,17) = 9.274, *p* = 0.007; famous faster than non-famous) and *Orientation* (F(1,17) = 36.217, *p* < 0.001; upright faster than inverted). Interestingly, there was a significant interaction effect (F1,17) = 6.155, *p* = 0.024), reflecting the advantage for familiar faces at upright orientation (196ms, i.e., an increase of 13.5% for unfamiliar faces), which is almost absent for inverted faces (62ms, increase of 2.8% for unfamiliar faces).

The second session confirmed these differences (Fig. 4A and B), with the inversion effect being slightly reduced overall (7.07 ± 5.56% inversion effect in accuracy, i.e., a 36% reduction; 363 ± 325ms in correct RTs, i.e., a 49% reduction). The statistical effects were confirmed (*Orientation* effect on accuracy scores: F(1,17) = 29.091, *p* < 0.001; *Familiarity* and *Orientation* effects on RT: F(1,17) = 24.062 and 22.406 respectively, *p* < 0.001 for both effects). The *Familiarity* x *Orientation* interaction effect on RT was only close to significance (F(1,17) = 3.649, *p* = 0.073). Indeed, while the advantage for familiar faces even increased for upright faces at testing session 2 (237ms, 18.4% of RT increase for unfamiliar faces), it also increased for inverted faces (130ms, 7.6% of RT increase for unfamiliar faces).

In short, the face identity matching task with these natural ambient images is performed almost perfectly by young adult participants, even for unfamiliar faces. While the face inversion effect is substantial for both familiar (12.5–6.5% decrease in accuracy for inverted vs. upright faces across sessions, 53.6%-32.4% increase in RT for inverted vs. upright faces) and unfamiliar faces (10.4–8.1% decrease in accuracy, 39.1%-20.3% increase in RT), both in accuracy rates and correct RTs, the advantage for familiar faces manifests essentially in terms of correct RTs for upright faces (-0.01 to + 0.51% inconsequential change in accuracy for unfamiliar vs. familiar faces, 13.5%-18.4% increase in RT for unfamiliar vs. familiar faces), with much less influence on inverted faces (2.4–2.2% decrease in accuracy, 2.8–7.6% increase in RT).

#### Test-retest reliability

We measured test-retest reliability both across participants (are participants’ accuracy scores and RT in each trial category similar from one session to another?) and across items (for each face item, are accuracy scores and RT similar from one session to another?) with Spearman’s correlation coefficients. Results can be found in Tables 3 and 4. Correlations showed high within-subject reliability between sessions for RT measures, but less so for accuracy, with only a non-significant trend for correlation for upright famous and inverted non-famous trials. Within-item reliability was high for both accuracy and RT, upright and inverted trials, with the exception of the RT correlation for non-famous upright trials not reaching significance.

**Table 3:** Within-subject reliability.

Across individuals (*n* = 18)	Fam Up	Fam Inv	Non Fam Up	Non Fam Inv
Correlation on accuracy scores	rho = 0.489 FDR *p* = 0.078	rho = 0.357 FDR *p* = 0.195	rho = 0.209 FDR *p* = 0.406	rho = 0.525 FDR *p* = 0.078
Correlation on RT	rho = 0.744 FDR *p* < 0.001	rho = 0.843 FDR *p* < 0.001	rho = 0.748 FDR *p* < 0.001	rho = 0.684 FDR *p* = 0.002

Spearman’s correlation coefficients between the first and second testing sessions were calculated for each familiarity and orientation type across all healthy participants (18 young). FDR-corrected p values associated with these correlation coefficients are displayed for each trial type.

**Table 4:** Within-item reliability.

Across items (*n* = 22)	Fam Up	Fam Inv	Non Fam Up	Non Fam Inv
Correlation on accuracy scores	rho = 0.653 FDR *p* = 0.002	rho = 0.556 FDR *p* = 0.009	rho = 0.664 FDR *p* = 0.002	rho = 0.471 FDR *p* = 0.027
Correlation on RT	rho = 0.784 FDR *p* = 0.002	rho = 0.921 FDR *p* = 0.002	rho = 0.350 FDR *p* = 0.111	rho = 0.518 FDR *p* = 0.02

Spearman’s correlation coefficients between the first and second testing sessions were calculated for each test measure and orientation type across the 22 items in each familiarity category. FDR-corrected p values associated with these correlation coefficients are displayed.

#### PS and age-matched controls

**Fig. 5:**
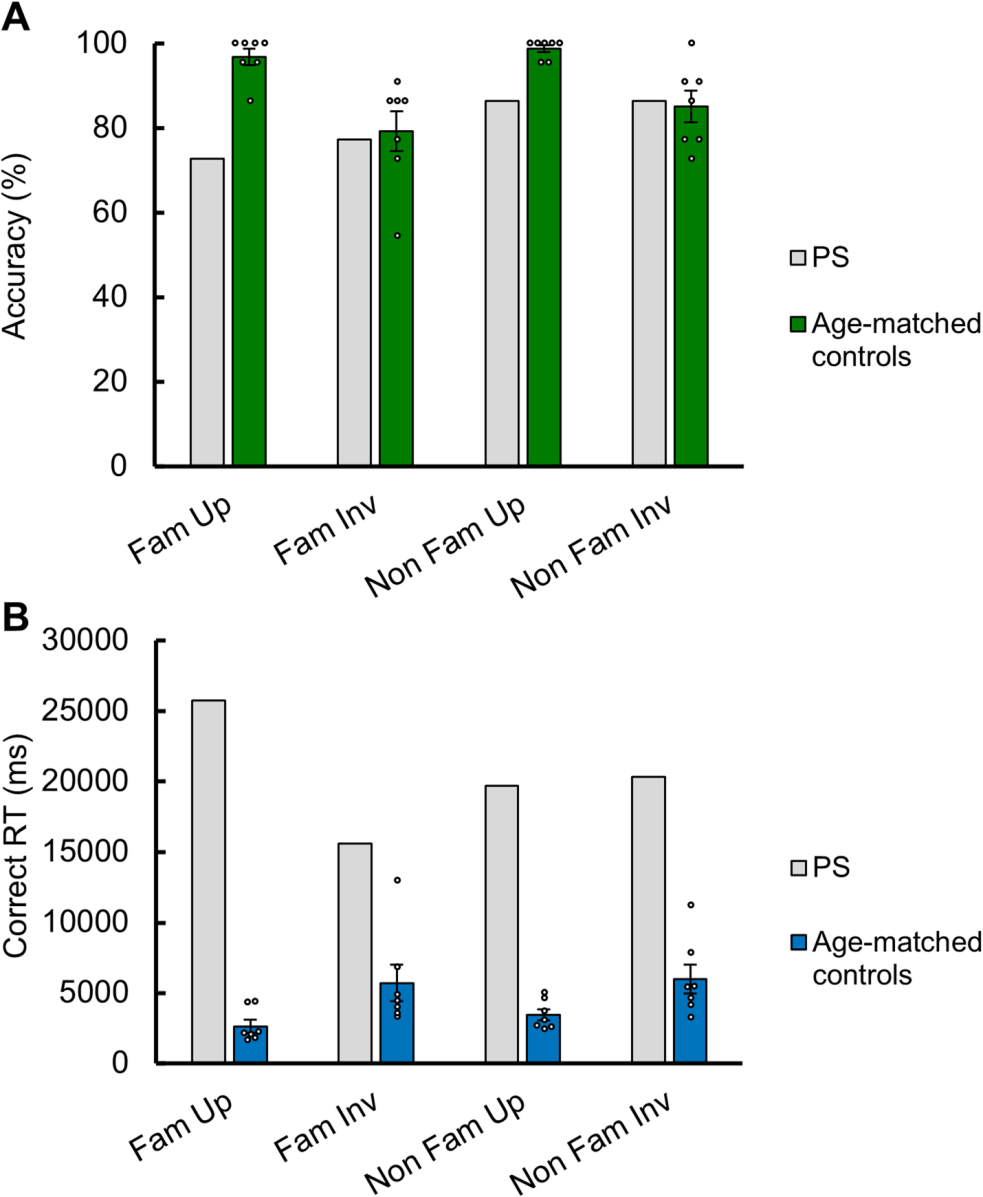
Mean accuracy (**A**) and correct response times (**B**) of PS and 7 age-matched control participants on the forced-choice face identity matching task. Black and white dots represent individual scores. Error bars represent standard errors.

Performance of PS and her age-matched controls is displayed in Fig. 5A for accuracy scores and Fig. 5B for correct RTs. PS’s overall performance in this task was of about 80%. While she scored below normal controls for upright faces, irrespective of familiarity, her accuracy rates were comparable to normal controls for inverted faces.

A two-way repeated measures ANOVA performed on accuracy scores on age-matched controls with *Familiarity* (famous, non-famous), and *Orientation* (upright, inverted) as main factors showed a significant effect of *Orientation* (F(1,6) = 15.094, *p* = 0.008). Neurotypical participants were on average 15.59% (± 10.6) more accurate for upright than inverted faces (range: 2.3–34.1% decrease in accuracy for inverted vs. upright faces), irrespective of face familiarity. There was also a significant effect of *Familiarity* (F(1,6) = 6.305, *p* = 0.046) but which can be attributed to a small advantage for unfamiliar inverted faces (79.2% accuracy for familiar vs. 85.1% for unfamiliar faces), performance being close to ceiling for upright faces (96.8% for familiar vs. 98.7% for unfamiliar faces) (Fig. 5A). Despite this, the interaction between the two factors was not significant (F(1,6) = 0.563, *p* = 0.481).

PS’s accuracy scores were well above chance (mean accuracy across trials = 80.7%, 18 correct items/22 possible items per trial type; vs. chance level at 50%, 11/22; χ^2^ = 4.956, *p* = 0.026). Yet, she scored lower than her controls on upright faces, regardless of familiarity (famous faces: t = 4.467, FDR-corrected *p* = 0.004; unfamiliar faces: t = 5.258, *p* = 0.004). Her accuracy scores were similar to controls for inverted faces (famous faces: t = 0.144, FDR-corrected *p* = 451; unfamiliar faces: t = 0.128, FDR-corrected *p* = 0.451) (Fig. 5A). Independently of familiarity, PS did not score higher for upright (M = 79.55%) than inverted (M = 81.85%) faces, i.e., she showed no inversion effect although her upright-inverted difference score was not statistically different from her controls (t = 1.576, *p* = 0.083). Independently of orientation, she did not score higher for famous (M = 75%) than non-famous (M = 86.4%) faces and there was only a trend to a significant difference with her controls for her familiar-unfamiliar difference score (t = 1.713, *p* = 0.069).

While age-matched controls were about two times slower than younger participants at this task, PS was extremely slow, taking close to 20 s on average to select one of the two face identities (Fig. 5B). She was systematically slowed down compared to age-matched controls, with her RT being on average 4.6 times slower (t range = 2.714–18.355, all FDR-corrected *p* < 0.05).

The statistical analysis performed on correct RTs of neurotypical participants showed a significant effect of *Orientation* (F(1,6) = 12.160, *p* = 0.013; slower for inverted than upright faces), with only a trend for a *Familiarity* effect (F(1,6) = 4.463, *p* = 0.079; faster for famous faces). Neurotypical older participants were on average 2802ms (± 2126) faster for upright than inverted faces (range: 972-7409ms advantage for upright vs. inverted faces). Despite a numerical face familiarity advantage for upright but not inverted faces (800 ms, 30.2%; inverted faces: 276ms, 4.8%), there was no significant interaction (F(1,6) = 1.955, *p* = 0.212).

Independently of familiarity, PS was not faster for upright (M = 22735ms) than inverted (M = 17980ms) faces, and her difference score (upright-inverted) was significantly different from the controls (i.e., faster for inverted; t = 3.325, *p* = 0.008). Independently of orientation, PS was not faster for famous (M = 20681 ms) than non-famous (M = 20034 ms) faces, again going in the opposite direction to what was observed in controls (i.e., faster for unfamiliar faces) although there was only a trend towards a significant difference in difference scores compared to her controls (t = 1.645, *p* = 0.076).

### Discussion

Despite being frequently exposed to these faces in real life (she watches TV, reads magazines displaying these faces, and can provide semantic information on these people if prompted with their name), PS never recognized any of the famous faces. In fact, despite spending tens of seconds on each triplet, she did not realize that there were famous people presented in the experiment. PS was able to perform the task well above chance level (about 80%), which is similar to her performance at (simultaneous or delayed) face identity matching tasks with (edited) pictures of unfamiliar faces (e.g.,^26,36,43,46^). She scored lower than age-matched controls but only for upright faces, also in line with previous evidence obtained with edited pictures of unfamiliar faces^36^. In the absence of time pressure, her difficulties at the task are better reflected in terms of correct RTs, with substantial response delays compared to age-matched controls for all conditions. While controls showed clear inversion effects and a trend for a face familiarity effect in RTs (for upright faces), PS showed no such effects at all. If anything, she was slightly better and faster for unfamiliar than familiar faces, which may reflect stimulus-driven differences in visual matching difficulty rather than a meaningful reversal of the familiarity effect (see general discussion). This interpretation is supported by the lack of significant famous > non-famous effect in controls.

## General discussion

Here we provide a validation of two simple nonverbal tasks of human face identity recognition (FIR) with natural images, these tasks having been introduced recently in the context of direct electrical stimulation (DES) to face-selective brain areas^24,25^. Even with a full set of items as used here, the tasks are easy to do for neurotypical (young or older) participants, leading to close to ceiling performance, and can be administered very rapidly (less than 5 min for task 1, 5–10 min for task 2 on average). Crucially, despite this easiness, these behavioral tasks are extremely difficult to perform for a well-documented case of prosopagnosia following brain damage, the patient PS. In task 1, the patient was unable to perform above chance level for selecting the familiar face identity among 3 face pictures, when normal controls were virtually flawless. In task 2, she scored at about 80% performance, which can be considered as very good but is nevertheless well below normal controls. Most importantly, this performance came at the expense of extremely (4–5 times) prolonged response times for the case of prosopagnosia (who does not show any slowing down in RT for matching non-face object shapes for instance^47^.

Thus, the contrast in performance between typical individuals and a well-validated abnormal FIR performer, which is key for proper neuropsychological evaluation (of this function)^7,48^, is maximized in these two tasks. In addition, the second task, requiring face identity matching across natural variations, is associated with clear effects of stimulus inversion and familiarity in normal participants. These two behavioral effects are arguably the most significant and documented effects in human FIR. Unsurprisingly, there was no effect of face familiarity in the case of prosopagnosia PS, who did not even notice that half of the trials were made of famous face identities. Also, PS showed no advantage whatsoever for upright over inverted faces, the latter observation being in line with previous evidence collected in this case and other brain-damaged patients with prosopagnosia (for evidence on PS and a review, see^36^, e.g.,^49–51^).

Importantly, at a more theoretical level, neurotypical participants’ performance at matching pictures of unfamiliar faces for their identity across substantial natural changes of viewing conditions in task 2 contradicts the often-stated view that human adults are ‘poor’ or ‘terrible’ at identity recognition of unfamiliar faces^29,52–58^. This view is largely based on the use of highly similar distractors and ambiguous tasks in terms of responses^10,29^, same/different tasks, matching tasks with unknown number of targets^54^, so that such tasks can only be easily resolved thanks to long-term semantic familiarity with the presented face identities. While such manipulations aim at maximizing the face familiarity effect, which is relatively modest here, they also create unrealistic non-ecological testing conditions for unfamiliar face identities. Here, while the face identity matching task with ambient natural images is not trivial, as demonstrated by the extreme difficulty for the case of prosopagnosia PS but also the large effects of face inversion (i.e., controlling for a matching based on low-level features), distractors are only roughly matched to target faces in terms of gender, age and obvious physical features (e.g., hair color) and the 2-alternative forced-choice task is unambiguous and not associated with response biases. Beyond demonstrating that even with natural ambient images neurotypical human adults are genuine experts at unfamiliar face recognition^59^, this simple testing mode provides crucial advantages in the context of neuropsychological testing of FIR. Below we summarize what we consider as advantages but also limitations and potential improvements/extensions of the two tasks validated here.

### Advantages of the tests

We think that the tasks described here present with several important strengths to evaluate human FIR integrity and ability.

First, their *validity*, i.e., measuring what it is supposed to measure, here the FIR function as it is in real life, has already been mentioned above. Task 1 requires to selectively match a sensory input among several possibilities to a visual representation in memory, while task 2 requires to both discriminate different identities based on their faces and to generalize across different natural views of faces, which is what FIR is about in real life. Pictures of faces are not edited, presented in full color, with all external features, different lighting conditions and background, etc., which is more ecological than in most if not all of FIR and famous face recognition tests and tasks reported in the literature (^8,9,17,23,60^, see Table 2 in ^7^ for review).

The (ecological) validity of the present tasks has already been shown to some extent in the two published DES studies, in which the patients were specifically unable or significantly impaired/slowed down at these tasks upon stimulation of key brain regions for FIR, in the lateral portion of the middle fusiform gyrus (‘fusiform face area’, FFA^25^) and the anterior fusiform gyrus^24^ of the right hemisphere. It is strengthened here by the two effects of familiarity and inversion of task 2. Admittedly, while we often have to recognize novel views of faces that we just encountered (i.e., novel faces) or we know (i.e., familiar faces) in real life, we rarely have to recognize inverted faces. However, regardless of its theoretical account (^34,49^, e.g., in terms of a disruption of feature-based processes or holistic/configural processes; see^61–63^ for reviews) the drop of FIR performance with stimulus inversion is extremely robust in neurotypical individuals, and arguably one of the most consistent effect observed across the board in experimental psychology. It is also important because there is exactly the same amount of sensory information between two faces to discriminate, or to match, at upright and inverted orientation. Yet, due to extensive life experience with upright faces and the right neural circuits, neurotypical human adults go beyond the sensory information provided to recognize upright faces better than inverted ones. Both effects, inversion and familiarity, are absent in cases of prosopagnosia such as PS^36^, but also in non-human species, in particular macaque monkeys (e.g.,^64,65^, for review, see^66^) so that they can truly be considered a hallmark of human (adult) expertise at this function.

Second, the test is highly *sensitive*, in the sense that inter-individual variability in the normal population is limited, with a high contrast in performance with the case of prosopagnosia tested. While it may be argued that the high contrast in performance observed here between PS and normal controls is simply due to the severity of the patient’s deficit, it is worth considering that this contrast is not so high for the same patient evaluated with all face identity recognition tests^48^. Asked to identify each familiar face of a known limited set for instance, she was able to succeed (with prolonged RTs) for 13 of the 27 individuals presented^43^, and it is only when mixing these same faces with unfamiliar faces and implementing unfamiliar/familiar decision tasks that her performance dropped almost to chance level^36^. Here, rather than asking whether a single face is familiar or not, task 1 creates a condition of uncertainty by adding two distractors who could equally well be the famous face identity, leading to chance performance for the patient.

As for face identity matching tasks, it is worth noting that PS’ score at the BFRT (39/54, 72.2%)^36^ for instance, ranks her only as ‘borderline’, with a highly significant difference with normal participants only when considering RTs (^36,67,68^, see also^51,69,70^ for other brain-damaged cases of prosopagnosia with borderline or even normal scores with only prolonged RTs). In the same vein, while PS was significantly impaired at the CFMT, her performance at that test (33/72) was only modestly contrasted to normal controls’ performance of the same age (Z = − 2.13, *p* < 0.05;^43^). Most recently, Fysh and Ramon (2022) showed that due to inadequate choices of stimuli, response biases and limited variables (i.e., lack of RT consideration), a series of face identity matching tests (GFMT, short version^10^, EFCT and PICT^13^) failed to identify PS as being impaired at FIR^48^. Finally, the addition of inversion and familiarity effects in task 2 here only serves to increase the test sensitivity in the sense that several abnormal patterns of performance can be detected in a tested individual.

Third, the evaluation is *fast*, the two tasks validated here being administered in a few minutes only in normal participants (about 10–15 min in total). This is a considerable advantage in the context of neuropsychological testing.

Fourth, the tasks are *simple* and *unambiguous* in terms of instructions and involve few cognitive/motor processes. In comparison, even the BFRT or its electronic version (BFRT-c) is heavier in terms of instructions and much harder in terms of spatial attention and short-term visual memory, with the requirement to select up to three face identities one after the other, such that certain categories of patient populations (e.g., with Alzheimer’s disease) are unable to complete the test (e.g., not being able to remember which face was already selected when making a choice between six, see^71^). Similarly, the CFMT, a popular FIR test, also requires the integrity of many FIR and non-FIR functions for learning and retrieving novel faces among multiple probes. In addition, it includes conditions that are not naturalistic (e.g., trials with added Gaussian noise) and difficult to the point that they do not help distinguishing normal from impaired performers^72^. The present 3-alternative (task 1) or 2-alternative (task 2) tests also allow to avoid any ambiguity as in same/different tasks (e.g., GFMT, EFCT, PICT) or tasks that require grouping face pictures based on a perceived identity (e.g., Facial Identity Card Sorting Test [FICST]^29^). They are also free of response biases since the correct face identity to select appears equally at one of the 3 (task 1) or 2 (task 2) positions.

Finally, the face identity matching task shows high test-retest *reliability* in response times across the four face conditions (range: Spearman’s rho = 0.68 to 0.84), which is comparable to what has been reported for the BFRT or CFMT (e.g.,^73–75^, for review see^7^).

### Limitations of the tests and potential extensions

Despite these strengths, the tasks validated here present also with a few disadvantages that should be fairly acknowledged.

First, they do not generate a large amount of inter-individual variability in accuracy rates, which would not make them ideal to study inter-individual variability in FIR in the normal population and identify potential cases of prosopdysgnosia/developmental prosopagnosia. It could be nevertheless informative to evaluate well-defined cases of prosopdysgnosia with such tasks to strengthen their validity (e.g., as in^76^).

Second, due to this ceiling performance in normal participants, test-retest reliability can only be assessed in terms of RT in task 2. While such RT measures are undoubtedly informative, they can also be misleading in the sense that many factors beyond the core FIR function can affect the time that an individual takes to respond at a given task (confidence, decision making, visual processing speed, etc.) (e.g., see^77^ for a study showing that, while BFRT-c accuracy did differentiate between people with developmental prosopagnosia and controls, RTs at this same task did not). In that sense, test-retest reliability should be better evaluated using the relative advantage in RT for upright over inverted faces.

Third, an obvious limitation is that the test requires to adapt and update pictures of famous faces to the population tested. Here the test is valid for a French/French Belgian population, as tested a few years ago, and famous people were selected with PS’ age range and culture in mind. The selection of famous identities (and level of familiarity with each of them) may vary considerably from one person to another, and the diversification of available media adds significant challenges for defining famousness, i.e., a face that may be perceived as unfamiliar by a person could be well-known to another. For these reasons, a cross-generational development/validation and regular updates of “familiar” vs. “unfamiliar” face databases are warranted. This is why the specific version of the test used in the present study is not presented online (in addition to copyright limitations) but versions following the same principles (i.e., natural images, forced-choice, assessment of face inversion and familiarity effects) could be developed to fit their targeted populations.

Fourth, a less obvious but important issue for task 2 is the difficulty of equalizing the ambient images, which differ in uncontrolled natural ways by definition, across items and conditions (familiar and unfamiliar) for matching difficulty. This may be one of the reasons why we did not observe a famous face advantage in accuracy for both younger and age-matched participants, although significant effects could be found in RTs for younger participants (trending towards significance for age-matched). Unexpectedly, a numerically reverse face familiarity effect (unfamiliar faster than familiar) was even observed in accuracy (mostly driven by inverted trials) in age-matched participants. This is likely to explain why PS performed better with unfamiliar face trials (Fig. 5), i.e., these trials were likely easier to match based on visual features, maybe due to closer resemblance between the triplets of images in familiar vs. unfamiliar trials or to different types of visual/configural differences between face identities to discriminate in familiar vs. unfamiliar trials. In this context, it could prove valuable to design and balance future FIR task trials based on well-validated impaired FIR performer cases who should, in theory, show no familiar face advantage (familiar = unfamiliar).

Fifth, another limitation is that no explicit familiarity checks were conducted in either task to confirm with participants that they were familiar with all famous identities. While this is unlikely to have affected the interpretation of PS’s results (given her complete lack of identity recognition), it could be a confounding factor in other cases, particularly for individuals with limited media exposure or cultural differences. This is less of an issue for the face and name familiarity selection tasks since the two modalities balance each other (i.e., if a participant does not find the famous name among distractors, they are also unlikely to recognize its face counterpart). However, this issue is more relevant for the face identity matching task, where familiarity effects are interpreted based on presumed recognition of famous identities. In future implementations, it would thus be beneficial to include post-task familiarity ratings to ensure that the familiar/unfamiliar distinction is valid for each participant.

In terms of extensions, the two tasks could benefit from the development of norms in a larger sample and more diverse sample, including participants across different age ranges. This would allow for a more precise evaluation of inter-individual differences, especially considering that performance patterns differed slightly between younger and older participants in our data (e.g., significant difference in RT for the face and name familiarity selection tasks in younger but not older participants). However, we emphasize again that the present study was not designed to establish normative benchmarks or diagnostic cutoffs. Instead, it serves as a proof of concept to demonstrate the potential of these two FIR tasks in clearly distinguishing between typical and impaired FIR performance, as illustrated by the contrast between neurotypical individuals and the case of PS. Note that any future attempt to establish norms should take into account the inevitable individual and cross-generational variations in familiarity with famous faces, as mentioned above.

The face identity matching task could also benefit from the implementation of limited time for exploring the face triplets, for example by adding a delay between the target face and the two probes that would discourage the use of analytical strategies. However, we would not recommend changing the time limit to respond as such time pressure has been found to have detrimental effects on face matching performance (e.g.,^78^).

It would also be interesting to assess the effect of contrast reversal on the performance at these two tasks. Indeed, while the detrimental effect of contrast reversal on FIR performance has been reported consistently along the face inversion effect (e.g.,^79–82^), it is rarely implemented in FIR tasks used in clinical contexts.

Finally, while behavioral tasks have unique advantages (e.g., they do not require a specific setup and can be conducted in various contexts including online), we remain convinced that implicit measures such as frequency-tagged EEG approaches with either edited (i.e., cropped^67^ or natural images (e.g.,^83,84^) may provide even more diagnostic measures in the future, letting behind the need for explicit instructions and difficult tasks to record objective and sensitive measures of FIR ability.

## Conclusions

In this paper we presented two novel FIR tests that were administered to younger and older neurotypical adult participants and the well-documented case of prosopagnosia PS. These tasks were designed to (1) assess famous identity recognition through faces and names with simple, straightforward instructions that do not require verbal output, and to (2) assess face identity discrimination through familiarity and orientation manipulations.

The results of normal participants contradict the view that humans are poor at unfamiliar face identity recognition tasks^29,52–58^, with performance close to ceiling for both familiar and unfamiliar faces in the face identity matching test that contains natural, non-edited images.

In addition, the results of the case of prosopagnosia PS validate these tasks as a sensitive assessment of FIR ability, showing (1) high contrast between impaired and preserved performance with faces and names, respectively, in the familiarity selection test, and (2) impaired performance with absence of face inversion and familiarity effects in the face identity matching test.

While limitations mentioned above, especially pertaining to the use of famous faces, prevent the copyright-free distribution of the version of the task used in the present study, and upon reasonable request, the authors are able to provide the stimuli and the codes to run these tasks, which would have to be adapted with pictures taken from the research team’s country/culture.

## Supplementary Information

Below is the link to the electronic supplementary material.

Supplementary Material 1

## Data Availability

The datasets used and/or analysed during the current study are available from the corresponding author on reasonable request.
