# Supplementary material for: A simple behavioral evaluation test of human face identity recognition with natural images validated with the case of prosopagnosia PS
Source: Sci Rep. 2025 Dec 4;15:43149. doi: 10.1038/s41598-025-27165-9 (PMC12678428; doi:10.1038/s41598-025-27165-9)
Supplement: Supplementary file 1 — Supplementary Material 1 [file 41598_2025_27165_MOESM1_ESM.docx]

**Supplementary Material**

**A simple behavioral evaluation test of human face identity recognition with natural images validated with the case of prosopagnosia PS**

**Supplementary Figures**


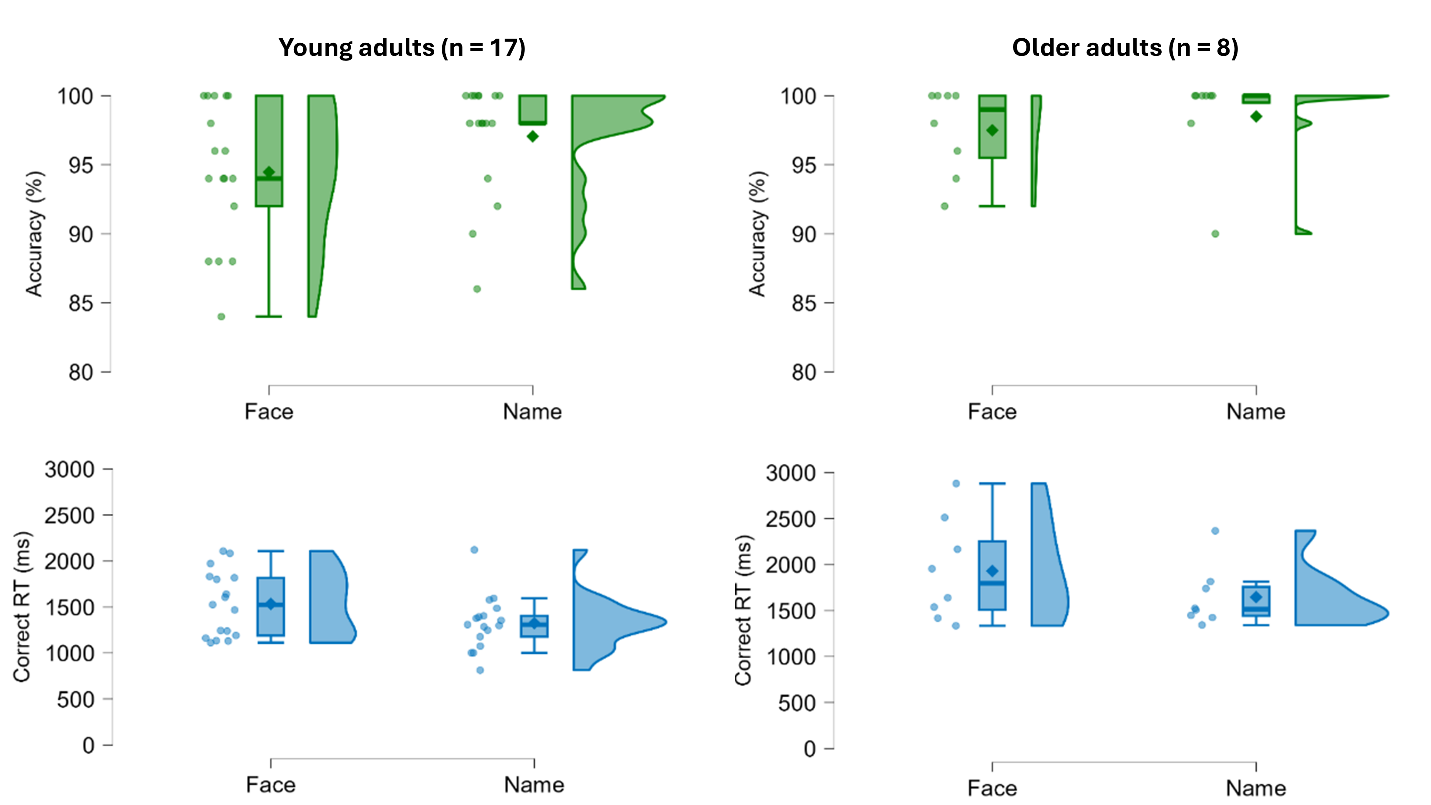


**Figure S1.** Raincloud plots showing individual data points, box-and-whiskers, and density distributions in the forced-choice familiarity selection task (accuracy on top, correct response times (RT) below) for young (n = 17) and older adults (n = 8). Diamond shape represents the mean in each condition (Face or Name). The same y-axis is used for young and older adults to facilitate comparisons between populations. Figures were created in JASP (version 0.95.1) (see Ott et al., 2025).


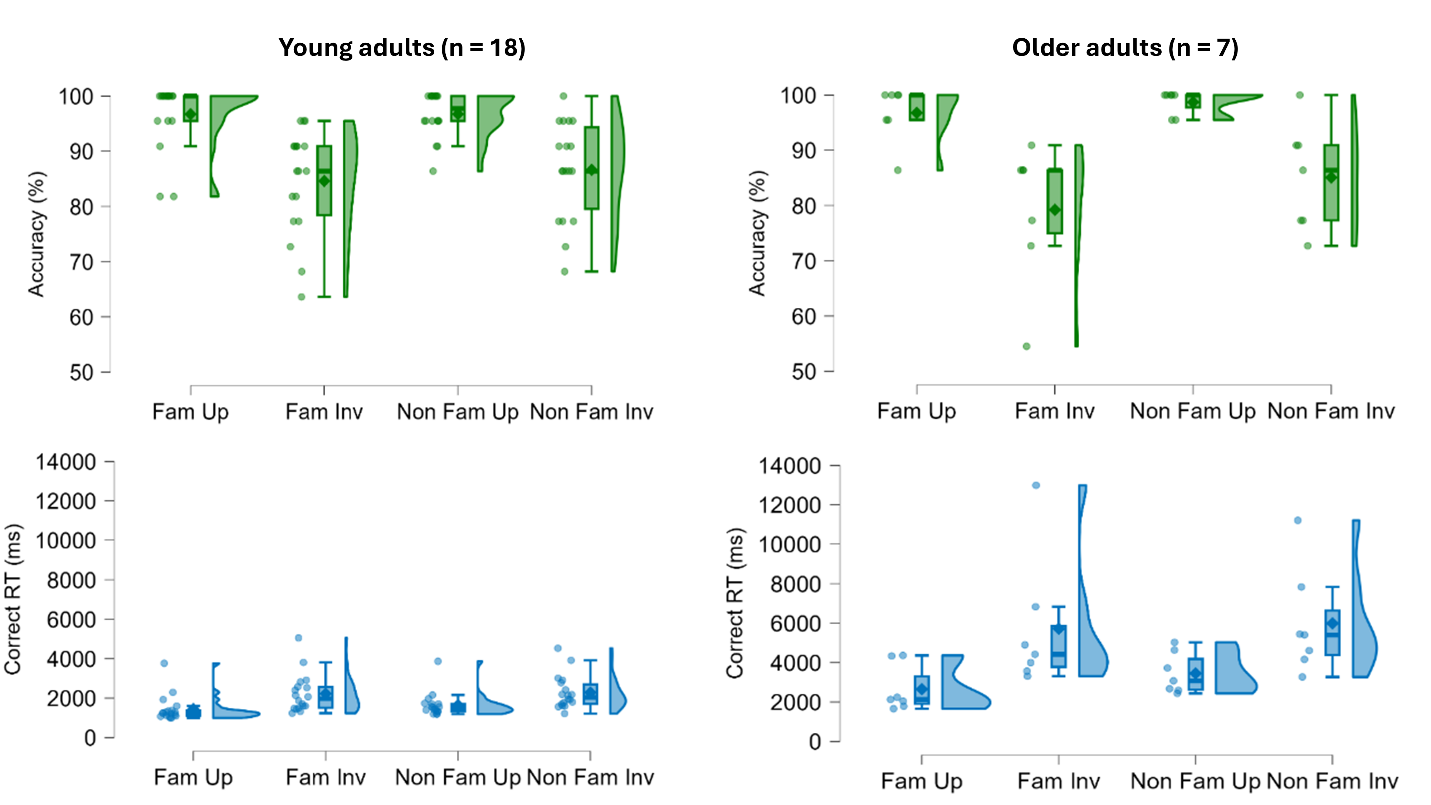


**Figure S2.** Raincloud plots showing individual data points, box-and-whiskers, and density distributions in the forced-choice face identity matching task (accuracy on top, correct response times (RT) below) for young (n = 18) and older adults (n = 7). Diamond shape represents the mean in each condition (Fam Up = Famous Upright, Fam Inv = Famous Inverted, Non Fam Up = Non Famous Upright, Non Fam Inv = Non Famous Inverted). The same y-axis is used for young and older adults to facilitate comparisons between populations. Figures were created in JASP (version 0.95.1) (see Ott et al., 2025).

**References:**

JASP Team (2025). JASP (Version 0.95.1) [Computer software].

Ott, V. L., van den Bergh, D., Boutin, B., van Doorn, J., Bartoš, F., Judd, N., van Langen, J., Korthals, L., Kievit, R., Groot, L., & Wagenmakers, E.-J. (2025). Informative data visualization with raincloud plots in JASP. *Behavior Research Methods*, *57*(9), 1‑11. https://doi.org/10.3758/s13428-025-02773-5

**Supplementary Tables**

**Table 1.** Accuracy and correct response times at the face and name familiarity selection task for each individual younger participant.

| Population | Subject | Age | Gender | Face Accuracy (%) | Face RT (ms) | Name Accuracy (%) | Name RT (ms) |
| --- | --- | --- | --- | --- | --- | --- | --- |
| Young Control | S01 | 24 | F | 98 | 1525 | 100 | 1354 |
| Young Control | S02 | 31 | F | 100 | 1134 | 100 | 1177 |
| Young Control | S03 | 25 | M | 84 | 2107 | 98 | 1594 |
| Young Control | S04 | 22 | F | 94 | 1468 | 92 | 1298 |
| Young Control | S05 | 23 | F | 88 | 1972 | 90 | 2121 |
| Young Control | S06 | 19 | F | 88 | 1818 | 94 | 1577 |
| Young Control | S07 | 22 | F | 92 | 1191 | 98 | 1002 |
| Young Control | S08 | 20 | M | 94 | 1640 | 100 | 1377 |
| Young Control | S09 | 22 | F | 94 | 1606 | 100 | 1075 |
| Young Control | S10 | 29 | F | 100 | 1162 | 100 | 1308 |
| Young Control | S11 | 23 | M | 94 | 1112 | 98 | 813 |
| Young Control | S12 | 24 | F | 100 | 1831 | 100 | 1486 |
| Young Control | S13 | 22 | M | 96 | 1239 | 86 | 1391 |
| Young Control | S14 | 25 | F | 96 | 1800 | 98 | 1404 |
| Young Control | S15 | 26 | M | 100 | 2083 | 98 | 1247 |
| Young Control | S16 | 21 | F | 88 | 1244 | 98 | 1285 |
| Young Control | S17 | 26 | M | 100 | 1131 | 100 | 1002 |

**Table 2.** Accuracy and correct response times at the face and name familiarity selection task for subject PS and each individual age-matched participant.

| Population | Subject | Age | Gender | Face Accuracy (%) | Face RT (ms) | Name Accuracy (%) | Name RT (ms) |
| --- | --- | --- | --- | --- | --- | --- | --- |
| Prosopagnosia case | PS | 65/66 | F | 36 | 13566 | 100 | 4381 |
| Older Control | S01 | 63 | M | 98 | 1538 | 100 | 1815 |
| Older Control | S02 | 65 | M | 100 | 1417 | 98 | 1448 |
| Older Control | S03 | 68 | F | 92 | 2511 | 100 | 1506 |
| Older Control | S04 | 63 | M | 94 | 2880 | 100 | 1524 |
| Older Control | S05 | 65 | F | 100 | 1954 | 100 | 1424 |
| Older Control | S06 | 68 | M | 100 | 1334 | 100 | 1343 |
| Older Control | S07 | 71 | F | 96 | 2166 | 90 | 2366 |
| Older Control | S08 | 70 | F | 100 | 1639 | 100 | 1739 |

**Table 3.** Accuracy and correct response times at the face identity matching task for each individual younger participant (first test administration).

| Population | Subject | Age | Gender | Fam Up Accuracy (%) | Fam Inv Accuracy (%) | Non Fam Up Accuracy (%) | Non Fam Inv Accuracy (%) | Fam Up RT (ms) | Fam Inv RT (ms) | Non Fam Up RT (ms) | Non Fam Inv RT (ms) |
| --- | --- | --- | --- | --- | --- | --- | --- | --- | --- | --- | --- |
| Young Control | S01 | 22 | F | 100.00 | 90.90 | 100.00 | 77.30 | 3764 | 5054 | 3866 | 4527 |
| Young Control | S02 | 23 | F | 100.00 | 95.50 | 95.50 | 95.50 | 1292 | 1597 | 1393 | 1562 |
| Young Control | S03 | 23 | F | 100.00 | 86.40 | 90.90 | 86.40 | 1045 | 2066 | 1187 | 2184 |
| Young Control | S04 | 26 | F | 100.00 | 90.90 | 100.00 | 90.90 | 1175 | 2133 | 1575 | 2419 |
| Young Control | S05 | 20 | M | 90.90 | 63.60 | 95.50 | 86.40 | 1234 | 1594 | 1705 | 1933 |
| Young Control | S06 | 23 | F | 95.50 | 81.80 | 100.00 | 68.20 | 1291 | 1472 | 1444 | 1762 |
| Young Control | S07 | 24 | F | 100.00 | 90.90 | 95.50 | 77.30 | 1091 | 1442 | 1303 | 1797 |
| Young Control | S08 | 24 | M | 95.50 | 86.40 | 100.00 | 77.30 | 1210 | 1868 | 1422 | 1691 |
| Young Control | S09 | 26 | M | 100.00 | 72.70 | 86.40 | 72.70 | 988 | 1234 | 1191 | 1215 |
| Young Control | S10 | 21 | M | 95.50 | 90.90 | 90.90 | 95.50 | 1073 | 1485 | 1325 | 1608 |
| Young Control | S11 | 20 | F | 81.80 | 95.50 | 95.50 | 95.50 | 1924 | 2530 | 1733 | 2133 |
| Young Control | S12 | 21 | F | 100.00 | 81.80 | 100.00 | 86.40 | 1241 | 2575 | 1532 | 2778 |
| Young Control | S13 | 24 | F | 100.00 | 86.40 | 100.00 | 86.40 | 994 | 1323 | 1185 | 1596 |
| Young Control | S14 | 31 | F | 100.00 | 95.50 | 100.00 | 90.90 | 1123 | 1711 | 1347 | 1923 |
| Young Control | S15 | 25 | M | 100.00 | 77.30 | 95.50 | 90.90 | 2290 | 2820 | 2156 | 3007 |
| Young Control | S16 | 22 | F | 100.00 | 77.30 | 95.50 | 95.50 | 1339 | 2396 | 1553 | 2174 |
| Young Control | S17 | 23 | F | 100.00 | 90.90 | 100.00 | 100.00 | 1376 | 2910 | 1699 | 2905 |
| Young Control | S18 | 19 | F | 81.80 | 68.20 | 100.00 | 86.40 | 1598 | 3806 | 1968 | 3914 |

**Table 4.** Accuracy and correct response times at the face identity matching task for each individual younger participant (second test administration).

| Population | Subject | Age | Gender | Fam Up Accuracy (%) | Fam Inv Accuracy (%) | Non Fam Up Accuracy (%) | Non Fam Inv Accuracy (%) | Fam Up RT (ms) | Fam Inv RT (ms) | Non Fam Up RT (ms) | Non Fam Inv RT (ms) |
| --- | --- | --- | --- | --- | --- | --- | --- | --- | --- | --- | --- |
| Young Control | S01 | 22 | F | 100.00 | 86.40 | 95.50 | 86.40 | 2983 | 2794 | 2975 | 2653 |
| Young Control | S02 | 23 | F | 100.00 | 100.00 | 100.00 | 86.40 | 1095 | 1389 | 1203 | 1548 |
| Young Control | S03 | 23 | F | 100.00 | 95.50 | 95.50 | 81.80 | 984 | 1802 | 1187 | 1517 |
| Young Control | S04 | 26 | F | 100.00 | 90.90 | 95.50 | 95.50 | 1249 | 1585 | 1481 | 1969 |
| Young Control | S05 | 20 | M | 100.00 | 86.40 | 100.00 | 86.40 | 1651 | 1859 | 1992 | 2352 |
| Young Control | S06 | 23 | F | 90.90 | 86.40 | 90.90 | 86.40 | 891 | 1056 | 941 | 1187 |
| Young Control | S07 | 24 | F | 100.00 | 95.50 | 100.00 | 72.70 | 871 | 1130 | 1203 | 1475 |
| Young Control | S08 | 24 | M | 100.00 | 95.50 | 100.00 | 86.40 | 1169 | 1695 | 1758 | 2050 |
| Young Control | S09 | 26 | M | 95.50 | 77.30 | 86.40 | 86.40 | 842 | 1209 | 1166 | 1089 |
| Young Control | S10 | 21 | M | 95.50 | 90.90 | 95.50 | 95.50 | 1143 | 1297 | 1431 | 1736 |
| Young Control | S11 | 20 | F | 95.50 | 90.90 | 95.50 | 90.90 | 1921 | 2174 | 2175 | 1978 |
| Young Control | S12 | 21 | F | 100.00 | 90.90 | 100.00 | 100.00 | 1126 | 1985 | 1401 | 2073 |
| Young Control | S13 | 24 | F | 100.00 | 81.80 | 100.00 | 81.80 | 830 | 1079 | 1079 | 1301 |
| Young Control | S14 | 31 | F | 100.00 | 95.50 | 100.00 | 100.00 | 955 | 1309 | 1196 | 1573 |
| Young Control | S15 | 25 | M | 100.00 | 86.40 | 100.00 | 90.90 | 1756 | 2254 | 1916 | 2481 |
| Young Control | S16 | 22 | F | 95.50 | 100.00 | 100.00 | 90.90 | 959 | 1470 | 1114 | 1247 |
| Young Control | S17 | 23 | F | 95.50 | 100.00 | 100.00 | 95.50 | 1274 | 1565 | 1396 | 1831 |
| Young Control | S18 | 19 | F | 90.90 | 95.50 | 95.50 | 95.50 | 1430 | 2974 | 1775 | 2894 |

**Table 5.** Accuracy and correct response times at the face identity matching task for subject PS and each individual age-matched participant.

| Population | Subject | Age | Gender | Fam Up Accuracy (%) | Fam Inv Accuracy (%) | Non Fam Up Accuracy (%) | Non Fam Inv Accuracy (%) | Fam Up RT (ms) | Fam Inv RT (ms) | Non Fam Up RT (ms) | Non Fam Inv RT (ms) |
| --- | --- | --- | --- | --- | --- | --- | --- | --- | --- | --- | --- |
| Prosopagnosia case | PS | 66 | F | 72.7 | 77.3 | 86.4 | 86.4 | 25745 | 15617 | 19725 | 20343 |
| Older Control | S01 | 68 | F | 95.50 | 90.90 | 100.00 | 100.00 | 4335 | 6829 | 4634 | 7832 |
| Older Control | S02 | 62 | M | 95.50 | 72.70 | 100.00 | 72.70 | 1658 | 4415 | 2670 | 4607 |
| Older Control | S03 | 71 | F | 100.00 | 86.40 | 100.00 | 90.90 | 2240 | 4894 | 3073 | 5442 |
| Older Control | S04 | 64 | F | 86.40 | 86.40 | 95.50 | 77.30 | 2043 | 3305 | 2587 | 3269 |
| Older Control | S05 | 68 | F | 100.00 | 86.40 | 100.00 | 86.40 | 2127 | 3567 | 3725 | 5402 |
| Older Control | S06 | 71 | F | 100.00 | 77.30 | 95.50 | 90.90 | 4362 | 12990 | 5023 | 11213 |
| Older Control | S07 | 71 | F | 100.00 | 54.50 | 100.00 | 77.30 | 1782 | 3992 | 2437 | 4161 |
